# The DPSIR Model for Environmental Risk Assessment of Municipal Solid Waste in Dar es Salaam City, Tanzania

**DOI:** 10.3390/ijerph15081692

**Published:** 2018-08-08

**Authors:** Emmanuel Kazuva, Jiquan Zhang, Zhijun Tong, Alu Si, Li Na

**Affiliations:** 1 School of Environment, Northeast Normal University, Changchun 130024, China; yim124@nenu.edu.cn or emmanuel.kazuva@out.ac.tz (E.K.); gis@nenu.edu.cn (Z.T.); alusi007@126.com (A.S.); lin152@nenu.edu.cn (L.N.); 2 Department of Geography, Open University of Tanzania, Box 23409, Dar es Salaam, Tanzania; 3 Key Laboratory for Vegetation Ecology, Ministry of Education, Changchun 130024, China; 4 GIS Laboratory, School of Environment, Northeast Normal University, Changchun 130024, China

**Keywords:** municipal solid waste, risk assessment, analytical hierarchy process, human health, Dar es Salaam city, urban environment, DPSIR model

## Abstract

Environmental risk has become an area of major concern and research, drawing special attention. This study on the environmental risk assessment (ERA) of Dar es Salaam Municipal Solid Waste comes at a time when the Government of Tanzania is becoming increasingly concerned about dealing with high levels of pollution from municipal solid waste (MSW). The paper employed the Driving force-Pressure-State-Impact-Response (DPSIR) model to establish an environmental risk indicator system and the analytical hierarchy process (AHP) to calculate and analyze risk values, based on the actual situation of MSW in the city of Dar es Salaam. It lists several measures that have been taken in response to the current significantly high levels of pollution, which have assisted in maintaining the environmental risk index (ERI) at a medium level (0.4–0.6) during the period from 2006–2017. However, these measures have not been adequate enough to manage the external pressure. The ERI has been increasing gradually, calling for timely formulation of demand-specific waste management policies to reduce the possibility of reaching the critical point in near future. With the use of the DPSIR model for ERA, this study has become highly valuable, providing empirical justification to reduce environmental risk from MSW, which is one of the main sources of environmental pollution in the urban areas of developing countries.

## 1. Introduction

The changes in human socioeconomic activities over recent decades have created remarkable changes in lifestyle and general consumption patterns. These activities have significantly contributed to the rapid increase in both volume and diversity of municipal solid waste (MSW) worldwide, making the available management approaches unable to keep pace with it [1]. The range of mismatch between MSW generation and its collection (or management) approaches is very wide. In this manner, MSW is predicted to be a major source of environmental pollution in large cities in near future [2,3,4,5]. The concern is relatively more serious in the urban areas of developing countries than in developed countries. This is because in developing countries it is further triggered by the rapidly increasing population and urbanization that has not been accompanied by the required expansion in basic services by the government as well as the public sector [5,6,7]. 

In Dar es Salaam, Tanzania’s largest commercial city, urbanization stands at 5.1%, and more than 70% of its dwellers live in slums and under-serviced settlements [8,9]. These areas are characterized by an extremely poor management of MSW due to multifarious factors, such as high levels of poverty, poor infrastructure (such as limited passable streets and roads), and inadequate waste management planning and approaches [10]. In nutshell, the city is facing a huge challenge in accommodating its rapidly increasing population, providing them with basic urban services, and enhancing economic growth and development, while ensuring environmental sustainability. 

### Management Systems of MSW in Dar es Salaam

For high-quality and sustainable MSW management services, a reliable management system is of paramount importance. Currently, all responsibilities for comprehensive waste management (ranging from management of collection points to that of the final disposal site) are distributed between the Dar es Salaam City Council (DCC) and the corresponding districts (Dar es Salaam’s Local Authority or DLAs). Each district (described in Section 2.1 below) is an independent local authority responsible for area cleaning, waste collection, transportation, fee collection at the household and the market level, and transportation of waste to the final disposal site; the DCC is responsible for providing waste treatment infrastructure. In fulfilling their responsibilities, the DLAs face several challenges, such as rapid population growth and the impacts of urbanization, political interference, and lack of necessary capacity for monitoring [11,12].

Mismanagement of MSW is now a global problem that reduces the quality of life in urban settings, across different continents [13]. Several studies on waste generation and management in urban settings indicate a close relationship between the population size of a particular area and the amount of waste generated [14,15]. However, there is a vast difference between the developed and developing countries in terms of their capacity to manage additional waste volumes. In developed countries, the additionally generated waste is matched by an advancement in management strategies and technologies. In the third world countries, while the population size and the amount of waste generated are increasing rapidly, improvement in management practices and systems is not evident [6]. For example, in Dar es Salaam, in 1988, when the city had a population of 1.4 million residents and the generated waste was approximately 1200 tons/day, the local authorities were capable of collecting around 58% of it. However, in 2012, when the city had 4.3 million residents and the generated waste amount rose to 4397 tons/day, the amount collected dropped to 44% [16]. Recent reports indicate that in 2014, when the city’s population was about 4.5 million and the amount of waste generated was 4605 tons/day, the collection was 42.7% [11,17]

Millions of people across the world make a living from the waste sector, mostly as waste pickers in the absence of a municipal governance solution. According to Medina [18], around 2% of the world’s population is dependent in some way or the other, on waste collection, processing, and recycling for earning its livelihood. Due to the waste management challenges in Dar es Salaam, approximately 60% of the generated waste remains uncollected [10,15,19]. As a result of the large amount of waste being uncollected and the available profit making opportunities through collecting, processing, and selling of waste, the informal sector plays a de facto central role in waste management in the city [15].

However, contribution from the private and/or the informal sector is not enough to resolve the municipal solid waste management (MSWM) problem in Dar es Salaam. This is because, the main motivation of informal waste collectors is profit making. Therefore, the collection by them is limited to waste items with a relatively higher market value [10]. In this sense, only a few items such as plastics, metal, and some type of cardboard attract the attention of informal waste collectors in Dar es Salaam city [15]. Therefore, the majority part of MSW with lower or considered to have no any market value remains unmanaged.

The report of the expert mission on integrated solid waste management (ISWM) for Dar es Salaam indicates that, currently, MSW generation rate stands at 0.82 kg/capita/day [11]. Therefore, while approximately 4740 tons/day of waste is generated, less than 2000 tons (35–40%) is collected [10]. Of the collected amount, only about 1200 tons of waste reaches the final disposal location at the Pugu garbage dump site (PGDS) [11]. Generally, the remaining amount of MSW is either not collected, or is burnt or haphazardly dumped in illegal areas (including open spaces such as streets, water bodies, beaches, and river banks); this results in harm to marine biodiversity, persistent floods due to blocked sewage systems [20], disease epidemics, and other health-related problems [21,22].

Several species of pathogenic bacteria, including Salmonella, Citrobacter, dysenteriae, freundii, *Citrobacter amalonaticus*, *Proteus vulgaris*, *Aerobacter aerogenes*, and *Klebsiella oxyotoca* have been identified in the solid waste in Dar es Salaam and other parts of the country [23]. Solid waste also attracts waste pickers, and animals, such as dogs, pigs, and rodents [24,25]. Further, studies have reported the presence of heavy metals in the solid waste, which, if present in higher than acceptable concentration, can impede the growth or affect the metabolism of cells of organisms, and can result in mortality of living organisms, including human beings [26,27]

True to the trend in most fastest-growing African cities, the population of Dar es Salaam doubles every 15 years [28]. The extrapolation from a report by the African Development Bank (ADB) shows that by 2030, Dar es Salaam will be the 5th largest city in the continent. Thus, by 2030, the MSW generated will exceed 7500 tons/day. Unless changes are made to the current management strategies, MSW will have serious implications for ecological, environmental, and human health in the near future [29]. Therefore, the environmental risk assessment of MSW is of paramount importance for sustainable decision making. This study considers MSW to be any waste material including household refuse, street sweeps, institutional waste, commercial waste, and construction and demolition debris discarded in urban areas that municipalities are responsible for managing through collection, transportation, treatment, and final disposal.

## 2. Material and Methods

### 2.1. Geographical Description of the Study Location

The study was conducted in Dar es Salaam, one of the oldest and largest towns in Tanzania that is presently the main commercial city. This city is located at 6°48′ S and 39°17′ E (−6.8000, 39.2833) on a natural harbor in the eastern coast of Africa and has sandy beaches in some areas. It covers an area of 1393 km^2^ and is divided into five administrative districts: Ilala, Kinondoni, Temeke, Ubungo, and Kigamboni. The latter two districts were formed recently in 2016 by splitting up Kinondoni and Temeke, respectively. For the sake of clarity and easy availability of the required data, the original three districts (Ilala, Kinondoni and Temeke) were considered for the study.

Ilala district forms the central business district (CBD) on the Oceanside known as ‘Posta’. It serves as a transportation hub for the city, as the Julius Nyerere-International Airport, the Central and Tazara-railway stations, as well as the Dar es Salaam port are within its boundaries. PGDS, the only formal dumpsite in the city is located to its west. Kinondoni is the northern district, and is the largest in terms of the population size. To the east of the city is Temeke, which is the largest district in terms of area. The city has more than 100 waste collection sites, ranging from skips to large open areas [11]; among these, only 13 are formal, offering services such as the monitoring of collection routines and maintenance. The remaining sites have less or no pick-up services and thus, have turned into illegal dumping sites. For primary information, a total of seven sites, including a formal and an informal site from each district, along with PGDS, the end point of disposal for Dar es Salaam MSW, were used for the study.

### 2.2. Concept of DPSIR Model

The study adopted the Driving force-Pressure-State-Impact-Response (DPSIR) model to assess the environmental risk from Dar es Salaam MSW. The model is derived from the Pressure-State-Response (PSR) framework, which was first proposed by the Organization for Economic Co-operation and Development (OECD) and the United Nations Environmental Programme (UNEP) in the late 1980s as a common framework for environmental evaluation. Under this framework, environmental problems and issues were taken as the variables based on the concept of causality-contending that, ‘’human activities exert pressure (P) on the environment and ecological systems that changes the state (S) of the natural environment. This provokes society’s response (R) to counter the changed natural environment’’ [30,31]. In its original form, the PSR framework has been criticized for being too linear, narrow, and so unidirectional that it does not clearly illustrate the forces behind the so-called pressure that changes the state of the environment. It neither indicates the direct or the indirect short- or long-term impacts of the changed environmental state [32].

Later on, in 1994 the OECD enhanced the PSR framework by adding driving force and impacts indices, forming the DPSIR model [33] and further adopted by the European Environmental Agency (EEA) in 1999, accommodating the new indices [34]. The DPSIR model therefore state that, ‘’Socioeconomic human development, commonly as driving force (D) exert pressure (P) on the environment, and as a result changes the beauty and natural forms of the environment (state) (S). The changed state constitutes impact (I) on the ecosystem and human health. These impacts provoke responses (R) from society to any of the index (driving force, pressure, state, or impact) through preventive, adoptive or curative measures’’ [30]. After being adopted by the EEA, this model has been used by several national and international organizations, including the United Nations (UN) in several environmental assessment and management initiatives, and by the United States Environmental Protection Agency (USEPA) in the sustainable Puerto Rico initiative. It has been a valuable tool for addressing and reporting the complex environmental issues related to human activities and the state of the environment [35,36].Thus, the DPSIR model has proved to be an effective tool for developing indicators and reporting the state of the environment and consequences of environmental degradation [31,37,38]. Further, it has been applied to urban planning and environmental impact assessment (EIA), land resource evaluation, the wetland ecosystem, health assessment, and other related areas [39,40,41,42,43].

### 2.3. Using the DPSIR Model to Establish the Evaluation Index System

The DPSIR model is a useful tool for deriving indicators for assessing and reporting the state of the environment for decision making. It was used in this study to develop a comprehensive environmental indicator system (CEIS) for analyzing environmental risk based on the actual state of MSW management in Dar es Salaam. Different from PSR framework, the DPSIR model has been confirmed to be descriptive and analytical enough, as it indicates a non-linear relationship between indices in a comprehensive manner, including the feedback loop during analysis.

**Figure 1:**
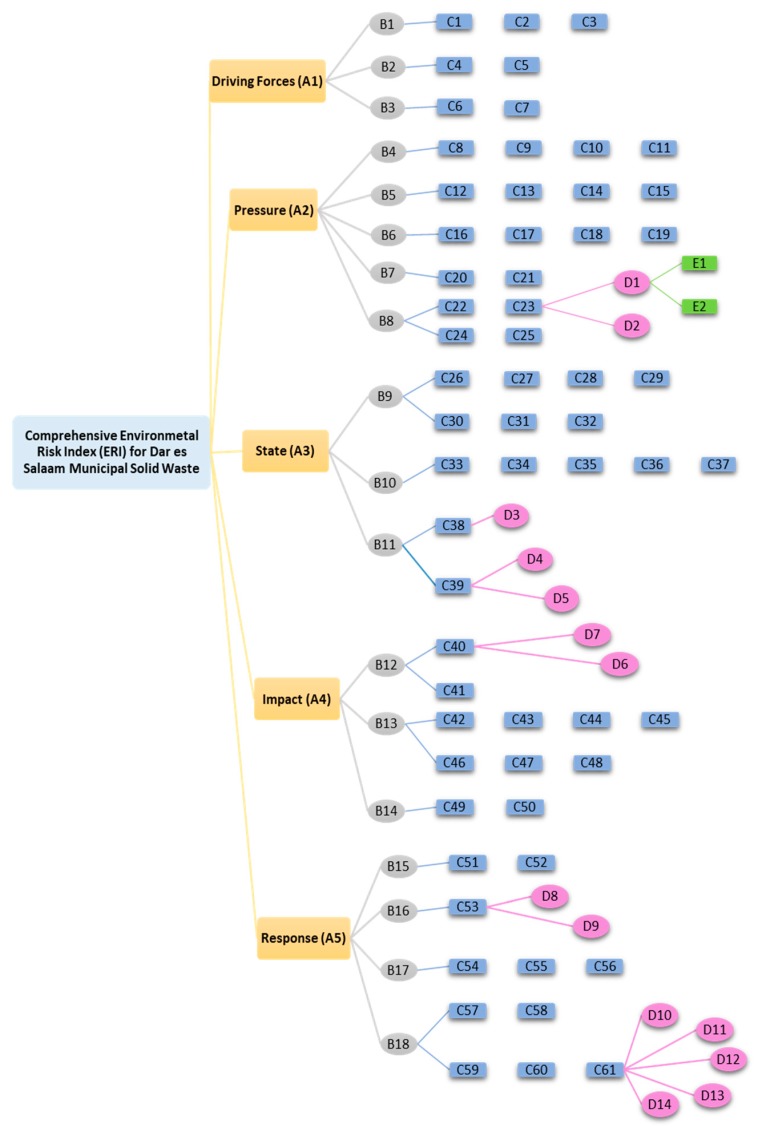
Hierarchical Organization of ERI system for Dar es Salaam MSW in DPSIR Model.

For ease of analysis, the environmental risk index (ERI) system was divided into three layers. The first layer is the evaluation elements layer or A-layer, which comprises five attributes of risk indices, labelled A_1_ to A_5_. The data indicators layer or the B-layer is the second layer, which consists of all the indicators used to assess the evaluation elements, labelled B_1_ to B_18_. The third is the risk target layer or the C-layer that consists of risk evaluation factors based on the actual waste management situation in the selected sites, labelled C_1_ to C_61_, along with other subordinates, D_1–14_ and E_1–2_ of the respective D and E sub-layers (see Figure 1 and the description in Appendix A). From this hierarchical arrangement, a critical analysis of the risk index from the evaluation elements layer in relation to the contents of two other layers was conducted.

### 2.4. ERI for Dar es Salaam MSW

*Environmental risk index* means the category of environmental risk used to calculate a fundamental beta, such as vulnerability, variability, and general probability in environmental risk analysis [44]. This study comprehensively analyzes the ERI for Dar es Salaam MSW, emphasizing mainly on the interrelationship between the prescribed indicators and actual situation in the area. The alternatives for decision making were measured and pairwise compared using the index in the evaluation elements layer. As shown in Figure 1 (and elaborated in Appendix A), the driving force (A_1_) involves factors that are crucial for the survival of human society, denoted by indicators B_1_, B_2,_ and B_3_. The three indicators have been drawn from Maslow’s work [45], well known as Maslow’s hierarchical model of human motivation that demonstrates life’s basic needs. The elements under this layer include food, shelter, water, healthcare facilities (HCFs), protection, family and community, and culture (C_1_–C_7_). The study considers the above as factors crucial for the survival of Dar es Salaam residents. However, any struggle and effort for meeting these basic needs puts pressure on the environment [46,47].

The pressure index (A_2_) refers to the direct human anger in response to the driving forces. It includes human engagement in different socioeconomic activities that consequently releases pressure resulting in waste generation. The corresponding indicators are shown in the B-layer (B_4_–B_8_), including building and construction (B_4_), population and society (B_5_), institutions and services (B_6_), energy and material consumption (B_7_), and the economy of the place (B_8_). The indicators are divided into 18 factors, C_8_–C_25_ under the C-layer with other subordinate factors in the D and E sub-layers (Figure 1, Appendix A).

The state index (A_3_) considers the existing situation of Dar es Salaam MSW. It focuses on the results of released pressure and the changed ‘state’ of environmental quality [48,49]. The ‘state’ may refer to only a natural system or to both natural and socioeconomic systems [34].

The theory behind the state index is that, in an effort to meet basic human needs, the quality of the natural environment is changed [50,51]. Therefore, the quality of various environmental compartments (soil, air, and water) is a function of the material released from the use of the respective compartments, the rate of use, and ability to be restored [21,52]. In other words, while people’s livelihoods and assets depend on their access to productive resources, the ability to control these resources is a part and parcel of environmental and human sustainability [53]. The ‘state of the environment’ is, therefore, a combination of physical, chemical, and biological conditions [54,55]. It can also refer to a wide range of features: qualitative and the quantitative characteristics of ecosystems, quantity and quality of resources, living conditions for humans, exposure to the effects of pressures on humans, and even larger socioeconomic issues. It is said that, the combination of the current state and the existing pressures explains the impacts [34,37].

This study considers the state index as the changed environment in Dar es Salaam resulting from pressure due to day-to-day human socioeconomic activities. Indicators under this index are organized into three major groups: B_9_, B_10_, and B_11_. As also indicated in Figure 1 and Table 1, they can be elaborated, as follows: B_9_ is the amount of waste generated from different sources (C_26–32_), B_10_ is the status of waste management, including total waste generated, amount collected and uncollected per year, and likewise (C_33–37_), and B_11_ is the level of pollution in the area in terms of observed land quality, settlement pattern, and water quality and direction (C_38–39_).

The study considers the following subordinates to the A_5_ index: B_15_, the institutional framework comprising institutional capacity, policies, law and regulations (C_51–52_); B_16_, education and publicity; B_17_, governance and investment (C_54–56_); and B_18_, application of modern approaches and technologies, as highlighted in the C-layer (C_57–61_). As indicated in Figure 1 and elaborated in Appendix A, A_5_ index is also subordinated by underlying factors from the D layer (D_8–14_).

### 2.5. Selecting Data Sources for the Study

The selection of the data was based on the main objective of this research, i.e., of assessing the environmental risk that arises from improper management of MSW. For effective environmental risk assessment (ERA) and prediction, knowledge about the actual situation of the MSWM in the study area is of vital importance [56]. Therefore, the data used for this study included the amount of waste generated and collected (Appendix B), major details of the waste management systems, such as infrastructure systems related to waste segregation, recycling, and risk prevention, protection, and mitigation measures. The remaining factors were human-based, such as the waste management experience and workers’ skills-sets from 2006–2017 (the risk assessment period (RAP)) [57].

The above data were procured from reliable sources, including the National Environmental Management Council (NEMC), the Vice President’s Office (VPO), and DLAs. Other information concerning the population and demographic trends which formed the basis for risk assessment and prediction, were obtained from the National Bureau of Statistics (NBS) (See Appendix A). The study also consulted experts in the field by using the experts’ questionnaire method (EQM) to generate views from experts and practitioners in the field. 

### 2.6. Environmental Risk Assessment for Dar es Salaam MSW

Risk assessment (RA) has become integral to various fields, such as business, ecology, and economics. More recently, it has been introduced in the field of environmental science, and is commonly termed as ERA. ERA refers to a scientific process that identifies and evaluates threats to the environment (comprising living organisms, their habitat and their ecosystem); this forms a part of the assessment of likelihood of potential hazards and their impacts, based on which risk mitigation measures can be undertaken [58,59] The ERA facilitates making of viable decisions for sustainable environmental management [60,61,62]. It has been confirmed a powerful tool for analyzing potential and extreme adverse environmental impacts, and has found to be with a wide application in decision-making processes [61].

### 2.7. Calculating ERI Using AHP and EQM

Calculation of ERI from a complex system and by the use of multi-classified factors, such as the one performed for Dar es Salaam MSW, is a difficult activity that requires development of calculation systems for sub-factors before a comprehensive ERI can be obtained; a single method or a combination of methods can be used [63,64]. The most common methods include the analytical hierarchy process (AHP), the fuzzy analytical hierarchy process (FAHP) technique, semi-structured decision-making approach, grey correlation, EQM, and scoring methods. In this study, a combination of two techniques (AHP and EQM) has been used [62,63].

The EQM was used for generating opinions from experts in the field. In this technique, a total of 38 questionnaires were distributed among local and international experts, practitioners, and professionals, particularly in the field of MSWM in urban settings. The level of feedback reached approximately 92%, confirming the experts’ questionnaires to be a valid method and the information obtained from it, worth using. The procedures for using AHP were followed (Section 2.7.1, Section 2.7.2, Section 2.7.3, Section 2.7.4 and Section 2.7.5), which enabled the computation of the ERI value from the variables in ‘A’ and ‘B’ layers.

The AHP was then employed for hierarchical analysis of the obtained index values, which helped to obtain the relative weights (average experts’ score) of the indicator system for Dar es Salaam MSW (see Table 2). This technique (AHP) introduced by Thomas Saaty in the 1970s that has been extensively studied and refined thereafter. The method is based on a mathematical and psychological analysis performed by combining subjective and personal preferences to organize risk factors in the RA process [63,65]. It is considered to be one of the best structural techniques to process personal, subjective preferences of individuals or groups, using pairs of relevant factors to assess and analyze risk for suitable decision making [61]. The major corresponding assumption is that, “the process of making a global or other widely relevant decision on complex tasks can be performed by separating and structuring a complex system into several simple tasks in the form of a hierarchical structure and making the pairwise comparison of the parameters” [66,67,68]. The AHP follows three major steps: computing vectors of criteria weights, computing matrices of option scores, and ranking options [66,69]

However, to simplify and make the process manageable, we divided the above three steps into five working steps: structuring the decision hierarchy, completing a pairwise comparison of options, checking the consistency of material judgement, computing the weights of each alternative with respect to the experts’ assessment, and aggregating the weights to determine the rank of each alternative for decision making [67,70]; the steps have been elaborated below. The combined use of AHP and EQM was, thus, relevant to this study, making it possible to quantify the weights of each index factor.

#### 2.7.1. Structuring the Decision Hierarchy

As presented in Section 2.2 and Section 2.3, the key indicators’ system for the comprehensive evaluation of ERI was established using six guiding principles proposed by Raybould [71]. According to Raybould, the selection of indicators system should follow the criteria, stated below:

- It should be based on the availability of accurate data; accuracy of data is the primary factor for correct analysis and decision making. As highlighted by Raybould, ‘’more accurate data enable more alternative/choices, which all together lead into better decision making”.
- It should focus on the scientific properties of the indicator objectives.
- It should be based on the principle of simplicity and lack of uncertainties and complexity.
- The established indicators system should be based on the rule of reliability, reflecting the real, existing environmental situation in the area.
- It should be fundamentally integral to the choice of the most suitable decision from the list of alternatives.
- It should be a useful tool for enlightening stakeholders on simplified scientific approaches to determine solutions based on the actual environmental conditions.

Based on these principles, the evaluation index system was selected to establish a comprehensive ERI for Dar es Salaam MSW.

#### 2.7.2. Assessment Scale and Construction of Pairwise Comparison Matrix

A statistical analysis was used to analyze the value of each risk factor from the obtained experts’ judgements score; this helped in the identification of comprehensive weights; (Weights in this case represent the relative importance of a particular indicator to the corresponding risk index for Dar es Salaam MSW) and ranks for each risk index in a hierarchical structure, as shown in Table 2.

For the most appropriate application of AHP, each cell in the matrix was assigned values using a numerical scale of 1 through 9 for each factor. The identified weights (*w*) (Table 2) were used significantly to analyze each parameter. Thereafter, a pairwise comparison of the parameters was done in the pairwise comparison matrix, highlighting the ratio of the identified indices (Table 3).

Pairwise comparison matrices for evaluation elements layer.

$$\left[\begin{array}{ccccc} 1 & 3/8.5 & 3/7.7 & 3/5 & 3/5.5\\ 8.5/3 & 1 & 8.5/7.7 & 8.5/5 & 8.5/5.5\\ 7.7/3 & 7.7/8.5 & 1 & 7.7/5 & 7.7/5.5\\ 5/3 & 5/8.5 & 5/7.7 & 1 & 5/5.5\\ 5.5/3 & 5.5/8.5 & 5.5/7.7 & 5.5/5 & 1 \end{array}\right]$$

The pairwise comparisons between two parameters, named parameter *i* and parameter *j*, in the evaluation elements layer, were made to assess their relative importance. Each point on the scale represents its own value and carries an important note for decision making. According to the linguistic judgement scale for interpreting results from a pairwise comparison matrix [65], the obtained numerical value (1–9) simply means that the two factors compared are correspondingly significant or that one is either more or less important than the other (Table 3).

The interim assessments were transferred, thereafter, to the objectives level, where the upper and higher level of the hierarchy determined the final aggregation of the estimates to obtain the rank, as indicated above in Table 2. The products of the comparison matrix for each row, which represent the value of a particular index compared to other indices in a comprehensive system, were computed using the following algorithm:

(1) $$\;M_{i}=\overset{n}{\underset{j=1}{\prod }}C_{ij}\;$$

Thereafter, the obtained products of the comparison matrix (*M_i_*) were computed to determine the normalized standard vector for the index (*V_i_*), using the following formula:

(2) $$\;V_{i}=\frac{\sqrt[{n}]{M_{i}}}{\sum_{i=1}^{n}\sqrt[{n}]{M_{i}}}\;$$

All indices, factors, and sub-elements contained in the different levels of the risk target layers in the system were calculated through the above processes, leading to results and final estimations. In this way, it was possible to interpret the risk level for each factor in the risk target layer.

#### 2.7.3. Measure of Consistency

Since experts’ assessments are based on human judgement, they are not exempt from personal preferences and subjectivity, increasing the possibility of creating uncertainties in drawing inferences from them. This is considered one of the challenges of using EQM, limiting the validity and reliability of the decision made from the obtained results [72,73]. Such kind of uncertainty is solved by performing a test of consistency. Test of consistency here refers to the measure researchers used to evaluate whether the relative judgement of the respondents were logically consistent or in need of review before further procedures [66].

In this study, the AHP method was used to test the consistency value of single factor from each of the levels. Using the aforementioned scale for the pairwise comparison matrix (1–9), the average random index (ARI) values were obtained. Saaty had earlier demonstrated that for a consistent reciprocal matrix, the largest eigenvalue (A value of a parameter for which a differential equation has a non-zero solution under the referred circumstance) should be equal to the size of the comparison matrix, i.e., *λ_max_* = *n*, where *λ_max_* is the maximum eigenvalue and n is the dimension (size) of the comparison judgement matrix [65]. Therefore, the measure of consistency, also known as the consistency index (*CI*), represents the deviation or degree of consistency at a single category or factor level. It is calculated using the following equation:

(3) $$CI=\frac{\lambda_{max}-n}{n-1}\;$$

Here, the *CI* indicates whether the judgements that are to be used for decision making, produce the desired reliable values in a set of evaluations.

Another common inconsistency in evaluation results may occur when a pairwise comparisons are performed for many index factors [72,73]. This type of inconsistency can be ascertained by checking the total, overall consistency using the consistency ratio (*CR*) from the obtained *CI*. In AHP, *CR* is defined as a comparison between the *CI* and random consistency index (*RI*) [72]. In its simple mathematical expression, this definition can be interpreted as:

(4) $$\;CR=\frac{CI}{RI}\;$$

Thus, the following equation illustrates the algorithm used to calculate the overall *CR*:

(5) $$\;CR=\frac{\sum_{i-1}^{n}a_{i}CI_{i}}{\sum_{i-1}^{n}a_{i}RI_{i}}\;$$

The toleration limit for observed inconsistency is valued at 0.1. The judgement responses in the pairwise comparison matrix can be considered to have acceptable consistency if the value for the *CR* is less than or equal to 10% [71]. If the *CR* exceeds this threshold. In this study, the obtained CR was below this threshold; thus, the values were acceptable.

#### 2.7.4. Relative Weights of Identified Risk Factors

Various factors identified to have been affecting the index system, whereby every evaluation element consisted of different risk factors as a subset of the comprehensive environmental risk index (CERI) system for Dar es Salaam MSW. Each subordinate for every index affects the system in different ways and so, it has its own impact. Due to the differences of their impact on ERI, it would be incorrect to evaluate and weigh them on an equal basis. Therefore, the set of factors corresponding to indicators at different levels of the hierarchy were considered differently. This was the main reason for employing the EQM. Table 4 present the obtained weight after computation of the risk weight as obtained using EQM.

#### 2.7.5. Standardizing Actual Data Scores and Identifying Risk Level

The standard scores indicated the deviation of the computed score/ERI from the data average. Standardization and dimensionless processing were important procedures for simplifying the evaluation index system of Dar es Salaam MSW, which constitutes multiple tasks with unified dimension. The data obtained were transformed into standard scores for each factor, of which, the standard data was between 0 and 1 for the positive and negative values of the indicators. Therefore, the following equations were used to obtain the standard scores:

(6) $$Z_{i}=\frac{x_{i}-x_{min}}{x_{max}-x_{min}}\;\;\mathrm{For}\;\mathrm{positive}\;\mathrm{values}$$

(7) $$Z_{i}=1-\frac{x_{i}-x_{min}}{x_{max}-x_{min}}\;\;\mathrm{For}\;\mathrm{negative}\;\mathrm{values}$$

From the obtained standard value of the actual data score, the evaluation score of the indicator (*S_i_*) was calculated, using the following equation:

(8) $$\;S_{i}=\overset{n}{\underset{j=1}{\sum }}X_{j}W_{j}\;$$

where, *S_i_* is the score of indicator (*i*), *Xj* is the value of *j* factor in *i* evaluation unit, *Wj* is the weight of the value of indicator *j,* and *n* is number of evaluation factors from evaluation element layer.

Finally, by using the standardized values of the actual data score and the evaluation score of indicator *j*, the ERI for the Dar es Salaam MSW was successfully calculated, as follows:

(9) $$\;ERI=\overset{5}{\underset{i=1}{\sum }}S_{i}W_{i}\;$$

Using the procedures above, the comprehensive ERI for Dar es Salaam MSW was calculated. The values and weights for all indicators were also identified.

For decision-making purposes, the risk values were grouped into five distinctive risk levels (RL). Each level (I–V) has different thresholds, with their lowest at 0.1–0.2 and highest at 0.8–1 [74]. Table 5 shows the classification of RL, remarks on pollution status, and the deemed ideal responses.

The classification of risk into these levels was highly useful for this study as it defined the exact level of risk related to Dar es Salaam MSW. The identified risk level provided a credible basis for recommending appropriate action towards sustainable environmental management in the urban areas, as per the actual situation in Dar es Salaam MSW [75].

## 3. Results

The ERI for Dar es Salaam MSW was found to be a function of different factors grouped into five major risk indices from the DPSIR model: the driving force, pressure, state, impacts, and response indices. These indices were subordinated using different indicators as subsets of a comprehensive ERI system, and were displayed as B- to E-layers (Figure 1 and descriptions in Appendix A). Each index affected the system in a different way and with a different magnitude, as presented in the following sub-sections.

### 3.1. Driving Force Index (A_1_)

**Figure 2:**
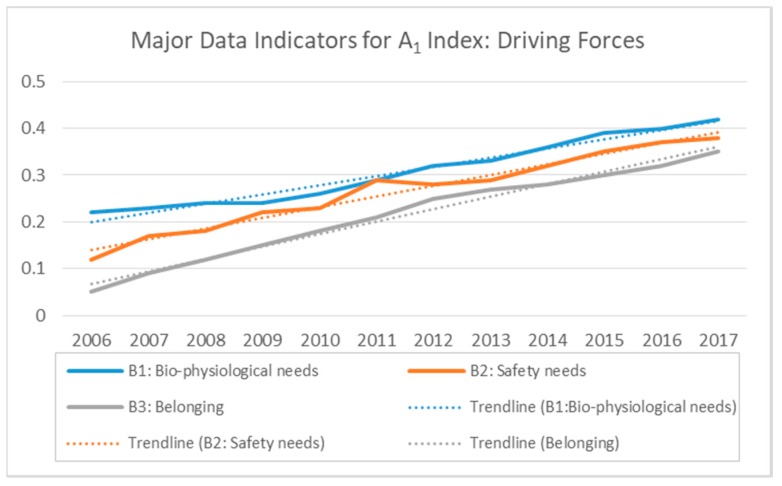
Major Data Indicators for A_1_ Index: Driving Forces.

The major data indicators for the driving force index (B_1_, B_2_, and B_3_) represent the factors essential for the survival of the Dar es Salaam community. These include B_1_ [food (C_1_), water (C_2_), and shelter (C_3_)]; B_2_ [health care facilities (HCFs) (C_4_) and protection from hostile environments (C_5_)]; and B_3_ [housing and safety needs (C_6_), and cultural factors (C_7_)]. The demand for these factors was found to be increasing with the increase in population size from 2006 to 2017, resulting in an increase of risk index, as well. In particular, the overall risk value for A_1_ index increased gradually from 0.1300 in 2006 to 0.3801 in 2017 (Figure 2). The index was found to have a significantly high impact on pressure (A_2_), resulting in an increased amount of Dar es Salaam MSW. However, this index with all its subordinate indicators had the lowest impact on the comprehensive ERI, with 0.3901 as the highest risk value in 2015, which falls under risk level II (0.2–0.4), i.e., a relatively low level. This necessitates the need to factor in the driving forces in any effort towards managing the urban environment, and MSW in particular.

### 3.2. Pressure Index (A_2_)

**Figure 3:**
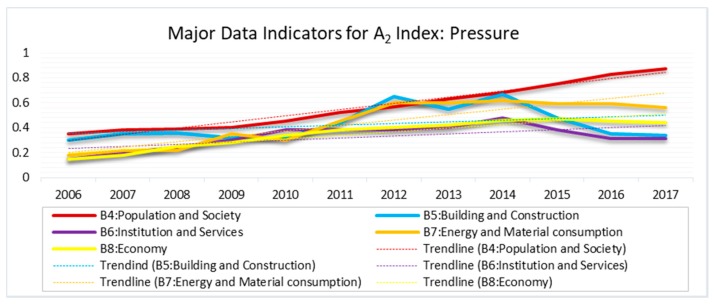
Major Data Indicators for A_2_ Index: Pressure.

The pressure index, subordinated by indicators B_4_ through B_8_, was demonstrated to have the greatest influence on the overall ERI of Dar es Salaam MSW. All the indicators in this index were in a continuous upward trend from 2006 to 2017 (Figure 3). Building and construction (B_5_) fluctuated considerably, especially between 2010 and 2016. It was a key factor in the rapid increase in MSW in 2012, though was slightly less significant than population and society (B_4_), in 2013. Both were almost equally significant in 2014. From 2015 to 2017, there was a prominent decline in risk value from building and construction, until it was below all other indicators, except the institutions and services (B_6_). The primary information reveals that the underlying reason behind this trend was the remarkable shift in people’s priorities away from building and construction; they started giving more attention to basic needs, such as paying for food, electricity, water bills and other basic services since the beginning of the term of the current government. Likewise, all other indicators, except population and society (B_4_), increased approximately interdependently until mid-2015, when the decrease in the risk index became apparent. Despite this decrease, each indicator registered a continuously increasing trend.

As Figure 3 indicates, population and society (B_4_) was found to have the greatest impact on the rapidly increasing MSW in Dar es Salaam. It was influenced by different subordinates under the C-layer, including population density (C_8_), population growth rate (C_9_), rate of urbanization (C_10_), and poverty ratio (C_11_). B_4_ increased significantly during the study period, with a minimum index factor of 0.3500 in 2006 and maximum of 0.8701 in 2017. This maximum value is greater than the critical point (0.800). However, the population living below the poverty line (C_11_) was one of the factors related to B_4_ that notably decreased between 2012 and 2017. This signifies an improvement in standard of living, which in turn, adds to the volume of waste generated. As pointed out by Hossain and others [1], the rate of waste generation is typically dependent on a country’s level of economy, standard of social services, and individual ability to access those services (wealth of an individual). This idea is consistent with the findings of this study that an increase in the per-capita income (GDP), which is a subordinate under B_8_ shown in Figure 3 above, has significantly influenced the additional volumes of waste generated, and the comprehensive ERI and risk level.

### 3.3. State Index (A_3_)

The state index represents the actual environmental conditions in Dar es Salaam with respect to MSW. This index is subordinated by waste generation rate, collection (management) rate, and pollution level, denoted by B_9_, B_10_, and B_11_, respectively.

**Figure 4:**
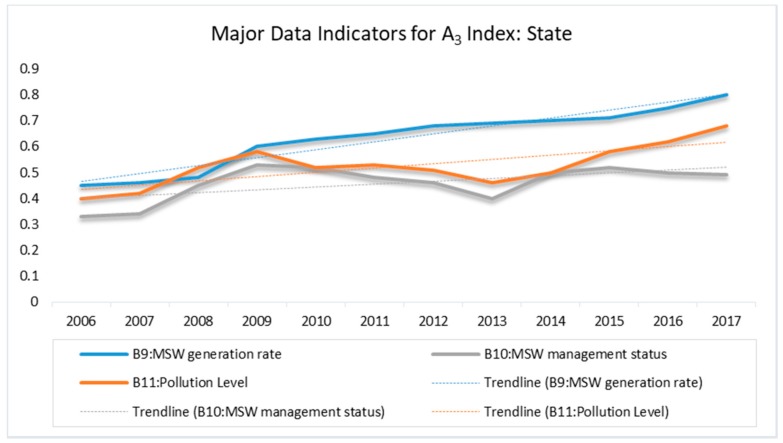
Major Data Indicators for A_3_ Index: State.

As indicated in Figure 4, the index value of the generation rate (B_9_) shows a continuously upward trend, particularly from 2008 to 2017. Substantial efforts were made to lower the high pollution level (B_11_). These efforts were reflected in the increasing amount of annual waste collection (B_10_), which resulted in a decrease in the index value, especially from 2010 to 2013 and from 2015 to 2017. However, these initiatives were not enough to suppress the existing pressure. The amount of uncollected and haphazardly dumped waste (C_36_) as well as the pollution level (B_11_) was high. The main reason for this failure was that, management status (B_10_) with all its variables (C_33–37_) were lagging far behind the rate of waste generation. For example, the maximum risk value for B_11_ (pollution level) reached 0.68 in 2017, which is under level IV [a relative high level (0.6–0.8)]. This signifies poor environmental quality, resulting from high external pressures that warrants serious measures to be taken before the extremely high level (level V), indicating a severe environmental damage, is reached.

### 3.4. Impact Index (A_4_)

**Figure 5:**
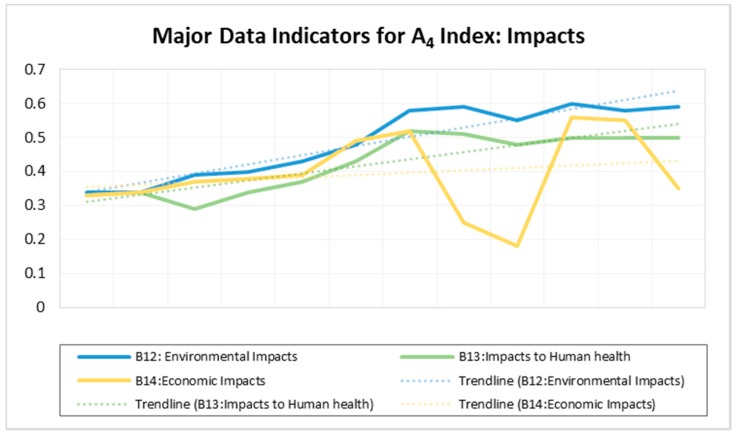
Major Data Indicators for A_4_ Index: Impact.

The impact index represents the effect of the inappropriate management of MSW in the city. Indicators of this index have been grouped into three categories, B_12_, B_13_, and B_14_ representing environmental impacts, human health, and economic impacts, respectively. The findings indicate that all these indicators registered a continuous upward trend for a period of 12 consecutive years (2006–2017) (See the trend lines in Figure 5). The environmental impacts (B_12_) were shown to have the highest risk index value (with an average of 0.4092), while human health and economic impacts registered average values of 0.3400 and 0.2505, respectively. B_12_ is basically influenced by the occurrence of environmental hazards (C_40_), such as persistent floods (as per the data for the period of twelve consecutive years, 2006–2017) (D_6_), odors and poor aesthetics (D_7_), destroyed ecosystem services (C_41_) such as poor climate regulation, and limited or unpleasant recreational areas.

Despite the upward trend displayed by all indicators of impact index as evident by Figure 5, there have been some fluctuations in the values for economic impacts (B_14_), especially from 2012 to 2017. The risk for B_14_ decreased to 0.18 in 2014, resulting mainly from several environmental campaigns, education and publicity, rigorous cleanliness, increased government funds for improving environmental quality, and the application of command and control (CACs) regulations in many parts of the city in 2013. Surprisingly, this trend did not have much influence on the indices for environment (B_12_) and health impacts (B_13_). They did not respond to the decreasing risk index for B_14_. Their weights in 2014 remained high at 0.59 and 0.48, respectively. This means that restoring the environment and human health from the impact of pollution requires a relatively long period of time, probably more than the time taken in destroying it.

### 3.5. Response Index (A_5_)

The response index stands for measures, plans, and approaches that are taken by government, private sectors, and community regarding the changed environmental state (A_3_) to reduce the impacts (A_4_) of pressure (A_2_) from driving forces (A_1_). This index assessed the efforts that have been made to reduce the level of vulnerability and individual exposure from polluted environment and the associated impacts. It was found that, A_5_ index have great influence on the risk value of other indices to the system. 

**Figure 6:**
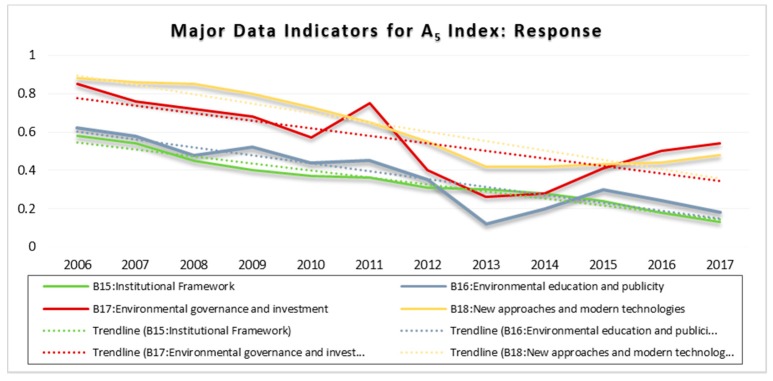
Major Data Indicators for the Response Index (A_5_).

All data indicators for A_5_ (B_15_–B_18_) showed a continuous or a fluctuating downward trend throughout the assessment period (see Figure 6). For instance, the downward trend for institutional framework (B_15_), and environmental education and publicity (B_16_) justify a continuous effort by the government, communities, and other stakeholders to increase institutional capacities, formulate well-defined policies, laws, and regulations, and raise awareness about sustainable MSW and general environmental management.

The observed fluctuation in B_16_ and B_17_ was mostly between 2010 and 2017, followed by an increasing trend level of risk for B_17_ and B_18_ between 2015 to 2017 at an average rate of 0.07 and 0.03, respectively. This indicates a low level of (or reluctance in) investing in MSW and related environmental projects (C_54–56_), including the application of modern technologies, comprising building of modern landfill sites (C_57_), recycling facilities (C_58_), and incineration plants (C_59_), as well as investing in waste-to-energy technologies (C_60_). Furthermore, low level of use and inappropriate application of new and modern economic instruments (EIs) (C_61_–D_10–14_) was also confirmed. Therefore, efforts made to suppress pressures and reduce the potential risk of MSW were inadequate for mitigating the problem. The findings was in consistent with the previous study [10].

**Figure 7:**
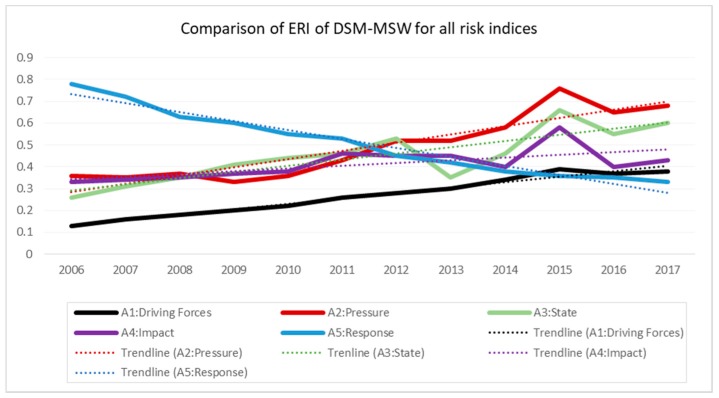
Comparison of ERI of Dar es Salaam MSW for all indices.

Generally, there is significant difference between A_5_ index and other indices (A_1_–A_4_). As shown in Figure 7, while the risk values for A_1_–A_4_ displayed a continuous upward trend, A_5_ showed a continuous downward trend from throughout the assessment period.

**Figure 8:**
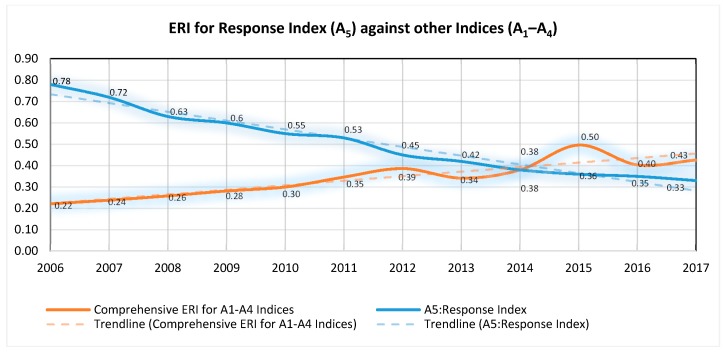
ERI for Response Index (A_5_) against other Indices (A_1_–A_4_) for.

Further analysis comparing the response index in one side and other indices indicates that, the risk values for the two groups are inversely proportional to one another; whereby the response index dictated this trend. In other words, any attempts made by the government or society as the response to the changed state of environment resulted in practical control and/or reducing the rate of other indices, particularly the external pressure. For example, in 2006 the society and government response to the changed environmental state was inadequate, and as a result, the risk values were considerably high (0.78), approaching to a critical point (0.78). The interpretation is that the ERI at that time was significantly influenced by the lack of proper response to the changed environmental quality and the impacts generated by the external pressure. Therefore, although the comprehensive ERI for other four indices (driving force, pressure, state and impact) was 0.22 (indicating a relatively low level of external pressure), the pollution level was high because the response was still inadequate. The value for these indices (A_1_–A_4_) gradually increased until 2012 where it was very close to the medium level (0.4–0.6). Despite the notable decrease in risk value for the response index from 2006–2017, the rate of decrease was not enough to immediately reverse the trend of ERI of other indices (A_1_–A_4_); making them in a continuously upward trend from 0.22 in 2006 to 0.43 in 2017. This clearly indicates that the forces from the response index (A_5_) index towards the system are not enough to suppress the trend of ERI. Therefore, for sustainable environmental risk reduction from MSW in the city of Dar es Salaam, special attention to response index is unavoidable. Figure 8 compares the trend for ERI of A_1_–A_4_ and the response index (A_5_).

### 3.6. Overall ERI for Dar es Salaam MSW

**Figure 9:**
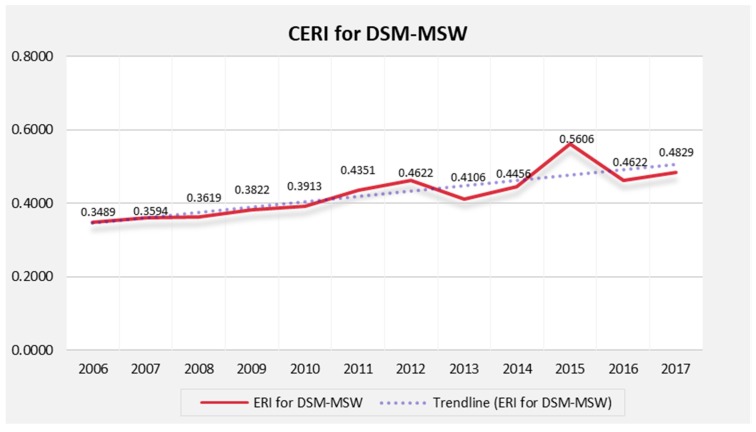
Comprehensive Environmental Risk Index for Dar es Salaam MSW.

The CERI for Dar es Salaam MSW from 2006 to 2017 was obtained using multiple methods. The trend line showed a continuous upward trend from its minimum value of 0.3489 in 2006 (see Figure 9). This value was within level II, suggesting a relatively low level of external pressures and signifying tolerable environmental conditions. However, in 2011, the ERI reached Level III with a value of 0.4351. This value corresponded to the medium level (0.4–0.6), indicating a substantial increase in the risk value due to external pressures. Remarkably, the change from level II to III happened within a period of less than five years, signifying an increase in risk index value by approximately 0.0172 per year. The index reached its maximum value (0.5606) in 2015, which was within level III or the medium level (0.40–0.60), though very close to level IV that is a relatively poor environmental state (Table 4).

The risk index value declined in 2013 and 2016 by 0.0516 and 0.0984, respectively. This decline suggested improved management approaches. In particular, the decline reflected responses from the government, communities, and other stakeholders through environmental management programs. For instance, during the same period, the institutional framework (B_15_), particularly the institutional capacities (C_51_) were improved, and environmental education and publicity (B_16_) were greatly emphasized; resulting into decline of risk value, and improved environmental quality (Figure 6).

However, due to factors mostly dominated by economic variables economic variables, this trend did not continue for long. There was a significant increase in the risk value in the years, 2014 and 2017 (Figure 9). Therefore, this trend cannot be considered as an actual decline of risk index value or a decrease in environmental pollution, but rather as a mere fluctuation in the trend. In addition, the final index values (2017) were still high, with all the features showing a continuous upward trend.

### 3.7. Major Contributing Indices

As indicated in Table 5, five levels have been used to categorize the ERI for Dar es Salaam MSW. From the given criteria, ERI is considered to be at an extremely high level (EHL) if it reaches level V (>0.8). Therefore, this study selected the comprehensive ERI of 0.8 as a critical point. The results presented above depict the state and weight of each index and Figure 9 presents the CERI for Dar es Salaam MSW from 2006 to 2017.

**Figure 10:**
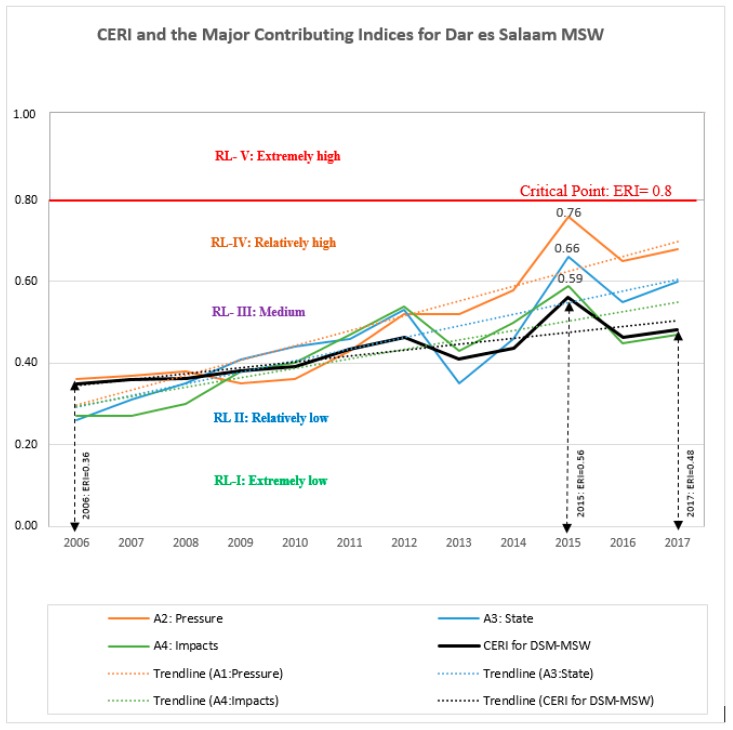
CERI and the Major Contributing Indices for Dar es Salaam MSW.

Even though each index potentially contributes to CERI, three major indices (pressure, state, and impacts) were identified to have the greatest influence on the current levels of environmental pollution for the city. For example, in 2015, the indices for pressure and state were >0.6, valued at 0.76 and 0.66, respectively. These values were both under level IV (0.6–0.8), representing a relatively high degree of risk. At the same time, the impact index was recorded at 0.59 (level III), falling within the medium level (0.4–0.6); which is very close to level IV, while the CERI was reading at 0.56. Therefore, calculations from the trend lines indicate that the three indices reach different levels of risk at different points of time, though earlier than CERI. Figure 10 presents the major contributing indices to the current pollution levels, and the CERI for MSW in the city of Dar es Salaam.

Therefore, the current (2017) position of CERI is under level III (medium level), valued at 0.48, which is an indication of the change in environmental state due to the external pressure (ref. Table 5). It is found that, three indices: Pressure (A_2_), State (A_3_), and Impact (A_4_) are the main contributing indices of the current environmental pollution level as they are almost at level IV (relatively high level). These indices are in upward trend, and are changing faster than the CERI. If not checked, these indices will reach the state of serious damage (critical point) in near future. These facts call for immediate responses to counter the situation by means of appropriate implementation of the response index (A_5_), giving special attention to pressure, state, and impacts indices.

## 4. Discussion

Improvement of available approaches and adaptation to newer technologies is an important aspect of sustainable waste management, as it reduces the potential risk to ecological environment and human health from mismanagement practices [76]. It needs to be mentioned that the Government of Tanzania has been experimenting with some approaches in the form of responses to reduce excessive pollution and bring about a change in the current environmental trend, especially in the city of Dar es Salaam [77,78]. However, these responses are weak and inadequate to deal with the actual level of pollution from MSW. There is little emphasis on the application of appropriate approaches such as EIs and adaptation to modern technologies, for the management of MSW in the city of Dar es Salaam [10].

As the results indicate, the influence of the pressure index on CERI is very high and is the main reason behind the poor environmental state of the city. As indicated in Figure 3, all factors for population and society (B_4_) greatly influence the increasing environmental risk from MSW. For example, urbanization rate in Dar es Salaam (C_10_) is very high, reflected by the increasing amount of waste generation which is approximately 5000 tons/day (Appendix B), with an annual increase of 10% [11]. Currently, no more than 40% of the generated waste is collected, while about 30% of it does not reach the final disposal site, the PGDS. The ERI is currently at a medium level, with an indication of continuous upward trend. Thus, there is no doubt that the ERI shows a rapid, upward trend, and the environmental state is degenerating alarmingly. If this trend is not reversed, soon, more people will be exposed to potential adverse environmental impacts from MSW.

The MSW management is predominantly the responsibility of the DLAs. However, due to the weaknesses in the current management systems, the private sector is participating indirectly in waste collection and management activities, mostly as informal scavengers who are collecting only the recyclable material with market value [10,15]. Engagement of private sector in MSW management is a positive development that can help resolve the problem. However, for this city, this sector is predominantly represented by informal scavengers whose only motivation is profit making; understandably, their major focus is on a small category of waste items with market value in the area.

In the absence of a proper waste segregation mechanism, the whole garbage is mixed up, leading to a higher risk to environment, service providers, and the public [79]. The mixed waste comprises a portion of usable materials such as waste paper, plastic, and some metals [80] that can be directly recycled. The direct and mixed disposal of all forms of waste not only results in significant wastage of resources, but also increases the volume of waste, thereby increasing the processing cost. Mixed waste contains hazardous material, such as fluorescent lamp and waste batteries. This increases the difficulty in its treatment while harming the ecosystem environment and human health [23].

With the rapid development of a country and the changes in life style of its people, a steep increase in the volume and diversity in the composition of MSW can be seen; thereby, investing on recycling facilities is unavoidable. Currently, the segregation facilities for waste treatment, reuse, and recycling are nearly non-existent. Recycling facilities exist for only certain types of waste, like glass, paper, cement, metals, and plastics; however, these cover less than 4% of the total waste that enters the system. Additionally, the segregation of materials for recycling is done by individuals with little or no relevant knowledge, and without proper equipment, making the process inefficient. Despite the several difficulties in waste collection and categorization in the city, waste recycling is highly important for the economic development and environment management as well as sustainability, warranting its efficient implementation of the process. It is evident that considerable scope for improvement exists in waste management strategy and planning, and in initiation of reuse, segregation, recovery and recycling of waste resources. In addition, the waste surveys can be conducted that will provide relevant scientific evidence and information for framing the waste disposal strategy as well as for setting the target in waste reduction.

The city has a reasonably big size for bringing cost-efficiency in its waste management system. Furthermore, the amount of waste generated is sufficient to run a waste-energy plant and/or a composting site [11,81]. This suggests that waste can be treated as a resource and an employment opportunity. This will be possible only if the appropriate policies are enforced and the proposed A_5_ index, with all its aspects (B_18–21_) is put comprehensively in place through public-private partnerships.

Results show an upward trend for ERI and that the risk to the city from MSW is increasing. If no measures are taken, the ERI will reach the critical point, (>0.8) in near future. This will be the point of EHL of pollution (level V), which is dangerous for ecosystems and human health. This warrant the need to make adequate efforts including the timely formulation of demand-specific waste management policies and implementation of measures, such as mass education to increase awareness, considering economic incentives, optimizing environmental infrastructure, improvising relevant laws and regulations, promoting waste reduction at source, implementing the policy of the Polluter Pays Principle (PPP) and all approaches, as indicated by C_61_ (D_10–14_).

Finally, the study recognizes the scope for improvement in the DPSIR model and the proposed indicator system. As postulated by Macgill and Siu [82,83] in the New Risk Theory, ‘the risk is dynamic’. In our study, the indicators in the system have a time aspect. For instance, the response index (A_5_) would be affected (delayed) by the publicity (B_16)_ and investment effect (B_17_) in a few years from now. For future research, it is suggested that time-related factors be introduced in the DPSIR model to enhance accuracy and timeliness of evaluation. This will accommodate the needs for the ERA based on spatial and temporal differences in waste management across the city, for providing credible information to the decision makers. 

## 5. Conclusions

This study aimed to assess the environmental risk posed by inadequate practices for MSW management in the city of Dar es Salaam, Tanzania. The AHP, EQM, and other relevant statistical methods have been used to analyze data from relevant sources.

The comprehensive ERI was successfully proposed in the study. This was done by means of categorizing the ERI system of the city into five sub-systems of drivers, pressure, state, impact, and response indices, based on the DPSIR model. Analytically, it can be understood that the ERI for Dar es Salaam MSW [ERI (DMSW)] is the function of the five major factors, which can simply be defined as; ERI(DMSW) = *f*(A1 + A2 + A3 + A4 + A5). The indices are subordinated by indicators from the B- to the E- layers. Although the indices affect the system in different ways, magnitudes and directions, their collective impact magnifies the pollution levels in the area. Results were drawn based on the calculation of the index value (weights), the element risk index for the driving forces, pressure, state, impacts, and response, and the ERI for the Dar es Salaam MSW for the period of 2006 to 2017. 

ERI for Dar es Salaam MSW displayed an upward trend and fluctuated in the study period; its lowest value was 0.35 in 2006, which was within level II (0.2–0.4), a relatively low level; and the highest value was 0.56 in 2015, which was within level III (0.4–0.6), the medium level, signifying rapidly increasing external pressure (ref. Figure 9 and Figure 10).

In general, in the study, the ERI for Dar es Salaam MSW for the study period was seen to fall under two distinctive groups: Level II, a relatively low level, during 2006–2010; and Level III, medium level for the period 2011 to 2017. However, both groups exhibited one commonality that the ERI showed a fluctuating yet increasing trend, year on year, and if not checked and trend reversed, the ERI will reach the critical point in near future preceded by pressure, state and impact indices.

With the application of the DPSIR model and quantitative AHP method, analysis of the environmental risk for Dar es Salaam MSW was made feasible. Immediate measures need to be taken in the form of practical application of the response index; particularly the timely formulation of demand-specific waste management policies such as the PPP and other relevant policy options, as indicated by C_61_ (D_10–14_).

Even though the study did not involve risk prediction, the comparison of the evaluation and analysis results for the study period with the actual situation of Dar es Salaam MSW reveals that the calculation and analysis made are rather accurate and reflects the actual MSW situation and several environmental and sanitation reports for the city.

The approach and methods used in this study can serve as references, and can be used for other cities of developing countries similar to Dar es Salaam, with a homogenous urbanization trend, with or without adjustment to the indicator system used in this study. The method used can also be used for analysis of MSW or other areas of environment analysis and risk prediction.

## Figures and Tables

**Table 1:** Environmental Risk indicator System for Dar es Salaam MSW.

**A1**	**Driving Forces**				
	**B1**	**Bio-physiological needs**			
		C1	Food		
		C2	Water		
		C3	Shelter		
	**B2**	**Safety needs**			
		C4	Healthcare		
		C5	Protection from hostile environment		
	**B3**	**Belonging**			
		C6	Need for Family and community		
		C7	Cultural practices		
**A2**	**PRESSURE**				
	**B4**	**Population and society**			
		C8	Population density		
		C9	Population growth rate		
		C10	Urbanization rate		
		C11	Population below poverty line		
	**B5**	**Building and Construction**			
		C12	Number of new buildings		
		C13	New built-up areas		
		C14	Total covered land		
		C15	Waste material generated		
	**B6**	**Institution and Services**			
		C16	Healthcare facilities (HCFs)		
		C17	Education services		
		C18	Transport and Communication		
		C19	Other Offices		
	**B7**	**Energy and Material consumption**			
		C20	Fuel		
		C21	Material use (e.g., building, etc.)		
	**B8**	**Economy**			
		C22	GDP per-capita		
		C23	Industries		
			D1	Services	
				E1	Hotels
				E2	Restaurants
			D2	Manufacturing	
		C24	Agriculture		
		C25	Markets (formal and informal)		
**A3**	**STATE**				
	**B9**	**MSW generation rate**			
		C26	Domestic waste		
		C27	Business and markets waste		
		C28	Water bodies and fishing garbage		
		C29	Waste from Healthcare facilities		
		C30	Construction and demolition		
		C31	Industrial waste		
		C32	Other major generates		
	**B10**	**MSW management status**			
		C33	Total waste generated/year		
		C34	Amount recycled		
		C35	Total amount disposed		
		C36	Amount left-over		
		C37	Annual tonnage of hazard waste Biohazard MSW (BhMSW)		
	**B11**	**Pollution level**			
		C38	Land pollution		
			D3	Settlement Pattern	
		C39	Water quality		
			D4	Toxicity level	
			D5	Direction of underground water	
**A4**	**IMPACTS**				
	**B12**	**Environment Impacts**			
		C40	Environmental hazards		
			D6	Persistent floods	
			D7	Odor and aesthetics impacts	
		C41	Ecosystem services (climate regulation and limited recreational opportunities)		
	**B13**	**Social Impacts (Human health-related impacts)**			
		C42	Malaria Vector		
		C43	Diarrhea		
		C44	Cancer		
		C45	Skin and respiratory diseases		
		C46	Eyes problems from uncontrolled burning		
		C47	Injuries for scavengers and children		
		C48	Deaths		
	**B14**	**Economic Impacts**			
		C49	Coast of abatement		
		C50	Economic repercussions		
**A5**	**RESPONSE**				
	**B15**	**Institutional framework**			
		C51	Institutional capacities		
		C52	Policies, Law and regulations		
	**B16**	**Environmental education and publicity**			
		C53	Promoting environmental management		
			D8	Rising public awareness	
			D9	Stakeholders’ involvement	
	**B17**	**Environmental governance and Investment**			
		C54	Funds for environmental project/s		
		C55	Enterprise environmental management		
		C56	Other environmental management expenses		
	**B18**	**New approaches and Modern technologies**			
		C57	Landfill		
		C58	Recycling		
		C59	Incineration		
		C60	Waste-to-energy technologies		
		C61	Application of Economic instruments (EIs)		
			D10	Polluter Pays Principle (PPP)	
			D11	Landfill tax	
			D12	Recycling credits	
			D13	Fee and charges	
			D14	DR-System and bond	

**Table 2:** Weights and rank of the evaluation elements (A-Layer).

Evaluation Element (Layer A)	Average Expert Score (*w*)	Rank
A1: Driving Forces	3.0	5
A2: Pressure	8.5	1
A3: State	7.5	2
A4: Impact	5.0	4
A5: Responses	5.5	3

**Table 3:** The judgement scale for pairwise comparison matrix.

Intensity of Importance	Linguistic Scale of the Pairwise Compared Parameter*s*, *i* and *j*	Description of the Status of the Compared Parameters
**1**	Equal importance/exactly the same	The two compared parameters contribute equally to the referred goal
**3**	Moderate/slightly importance	Experience and judgement slightly favor one parameter over another
**5**	Strong/serious importance	Experience and judgement strongly favor one parameter over another
**7**	Very strong/more serious importance	One element is favored very strongly over another, and its domination is demonstrated in practice
**9**	Extreme/absolute importance	The evidence favoring one parameter over the other is of the highest possible order of confirmation
**2, 4, 6, 8**	Intermediate value/the same importance	The referred elements have nearly equal importance

**Table 4:** Weights for ERI for Dar es Salaam MSW.

Evaluation Elements (A-Layer)			Data Indicators (B-Layer)		
Evaluation Index	Average Experts’ Score	Weight (w)	Data Indicator	Average Experts’ Score	Weight (w)
A1: Driving Forces	3.0	0.157	B1: Bio-physiological needs	6.5	0.4333
			B2: Safety needs	5.0	0.3333
			B3: Belonging	3.5	0.2332
A2: Pressure	8.5	0.447	B4: Population and Society	9.0	0.2267
			B5: Building and construction	8.5	0.2144
			B6: Institution and services	7.5	0.1889
			B7: Energy and material consumption	7.7	0.1933
			B8: Economy	7.0	0.1767
A3: State	7.5	0.354	B9: MSW generated rate	9.0	0.3745
			B10: MSW management status	8.5	0.3544
			B11: Pollution level	6.5	0.2702
A4: Impacts	5.0	0.285	B15: Environment impacts	9.0	0.4092
			B16: Human health impacts	7.5	0.3400
			B17: Economic Impacts	5.5	0.2505
A5: Responses	5.5	0.314	B18: Institutional framework	8.7	0.2626
			B19: Environmental education and publicity	7.5	0.2372
			B20: Environmental governance and investment	7.0	0.2200
			B21: New approaches and Modern technologies	8.5	0.2787

**Table 5:** Classification of risk level and interpretation guide

Risk Level	Value (Weight)	Degree of Risk	State	The Ideal Required Action
I	0.1–0.2	Extremely low	Low external pressure	Good condition, needs to be maintained
II	0.2–0.4	Relatively low	Less external pressure	Good condition, vigilance required to avoid further disturbances
III	0.4–0.6	Medium	Environmental state is changing with external pressure	Need to work on the changing state
IV	0.6–0.8	Relatively high	Poor state with large external pressure	Immediate action and management programs required at all levels of the system (DPSIR)
V	0.8–1.0	Extremely high	Serious damage due to great pressure	Dangerous environment for animals and human living; rehabilitation programs are urgently required

## Appendix A

**Table A1:** Environmental Risk Indicator System for Dar es Salaam MSW and Data Sources.

Index						Major Data Input	Major Data Source
**A1**	**Driving Forces**						
	**B1**	**Bio-physiological needs**				Both, qualitative and quantitative, primary and secondary data, regarding drivers for survival of the Dar es Salaam Community	Field Survey, 2016/2017
		C1	Food				
		C2	Water				
		C3	Shelter				
	**B2**	**Safety needs**					
		C4	Healthcare				
		C5	Protection from hostile environment				
	**B3**	**Belonging**					
		C6	Need for Family and community				
		C7	Cultural practices				
**A2**	**PRESSURE**						
	**B4**	**Population and society**				Secondary quantitative data on population	National Bureau of Statistics (NBS)
		C8	Population density				
		C9	Population growth rate				
		C10	Urbanization rate				
		C11	Population below poverty line				
	**B5**	**Building & Construction**					
		C12	Number of new buildings			Both, qualitative and quantitative, primary and secondary data	Field Survey, 2016/2017
		C13	New built-up areas				
		C14	Total covered land				
		C15	Waste material generated				
	**B6**	**Institution & Services**					
		C16	Healthcare facilities (HCFs)			Both, qualitative and quantitative, primary and secondary data on institutional and service waste	Field Survey, 2016/2017
		C17	Education services				
		C18	Transport & Communication				
		C19	Other Offices				
	**B7**	**Energy & Material consumption**					
		C20	Fuel			Both, qualitative and quantitative, primary and secondary data	Field Survey, 2016/2017
		C21	Material use (e.g., building, etc.)				
	**B8**	**Economy**					
		C22	GDP per-capita			Trend for all years	NBS, BOT (Bank of Tanzania)
		C23	Industries				Field Survey, 2016/2017
			D1	Services			
				E1	Hotels		
				E2	Restaurants		
			D2	Manufacturing			
		C24	Agriculture				
		C25	Markets (formal &informal)				DLAs
**A3**	**STATE**						
	**B9**	**MSW generation rate**					
		C26	Domestic waste			Both, qualitative and quantitative, primary and secondary data	Field Survey, 2016/2017; DLAs
		C27	Business and markets waste				
		C28	Water bodies and fishing garbage				
		C29	Waste from Healthcare facilities				
		C30	Construction and demolition				
		C31	Industrial waste				
		C32	Other major generates				
	**B10**	**MSW management status**					
		C33	Total waste generated/year			Both, qualitative and quantitative, primary and secondary data	DLAs, Secondary sources cited, Field Survey, 2016/2017;
		C34	Amount recycled				
		C35	Total amount disposed				
		C36	Amount left-over				
		C37	Annual tonnage of hazard waste Biohazard MSW (BhMSW)				
	**B11**	**Pollution level**					
		C38	Land pollution			Both, qualitative and quantitative, primary and secondary data	Field Survey, 2016/2017; DLAs, Secondary sources cited
			D3	Settlement Pattern			
		C39	Water quality				
			D4	Toxicity level			
			D5	Direction of underground water			
**A4**	**IMPACTS**						
	**B12**	**Environment Impacts**					
		C40	Environmental hazards			Both, qualitative and quantitative, primary and secondary data	Field Survey, 2016/2017; DLAs,
			D6	Persistent floods			
			D7	Odor and aesthetics impacts			
		C41	Ecosystem services (climate regulation & limited recreational opportunities)				
	**B13**	**Social Impacts (Human health-related impacts)**					
		C42	Malaria Vector			Both, qualitative and quantitative, primary and secondary data	MNH (Muhimbili National Hospital), Field Survey, 2016/2017; DLAs,
		C43	Diarrhea				
		C44	Cancer				
		C45	Skin & respiratory diseases				
		C46	Eyes problems from uncontrolled burning				
		C47	Injuries for scavengers& children				
		C48	Deaths				
	**B14**	**Economic Impacts**					
		C49	Coast of abatement				DLAs, NBS, BOT
		C50	Economic repercussions				
**A5**	**RESPONSE**						
	**B15**	**Institutional framework**					
		C51	Institutional capacities			Information on the available institutional capacity for Environmental monitoring and management	NEMC, VPO, DLAs
		C52	Policies, Law & regulations				
	**B16**	**Environmental education & publicity**					
		C53	Promoting environmental management			Both, qualitative and quantitative, primary and secondary data	VPO, DLAs
			D8	Rising public awareness			
			D9	Stakeholders’ involvement			
	**B17**	**Environmental governance & Investment**					
		C54	Funds for environmental project/s			Information on the available projects and budget for Environmental monitoring and management	VPO
		C55	Enterprise environmental management				
		C56	Other environmental management expenses				
	**B18**	**New approaches &Modern technologies**					
		C57	Landfill			Information on the available approaches and technology used for Environmental monitoring and management	Field Survey, 2016/2017 VPO
		C58	Recycling				
		C59	Incineration				
		C60	Waste-to-energy technologies				
		C61	Application of Economic instruments (EIs)				
			D10	Polluter Pays Principle (PPP)			
			D11	Landfill tax			
			D12	Recycling credits			
			D13	Fee and charges			
			D14	DR-System and bond			

## Appendix B

**Table A2:** MSW Management Status in Dar es Salaam (2006–2017).

Year	District	Average Amount of Waste (Tons/day)			
		Generated	Collected	Disposed at PGDS	Uncollected
2006	Kinondoni	2003	745	484	1258
	Ilala	1024	381	248	643
	Temeke	903	336	218	567
	**Total**	**3930**	**1462**	**950**	**2468**
2007	Kinondoni	2084	775	504	1309
	Ilala	1117	416	270	701
	Temeke	914	340	238	574
	**Total**	**4115**	**1531**	**1072**	**2584**
2008	Kinondoni	2101	782	547	1319
	Ilala	1207	449	314	758
	Temeke	978	364	255	614
	**Total**	**4286**	**1594**	**1116**	**2692**
2009	Kinondoni	2243	834	584	1409
	Ilala	1261	469	328	792
	Temeke	950	353	247	597
	**Total**	**4454**	**1657**	**1160**	**2797**
2010	Kinondoni	2230	830	626	1400
	Ilala	1240	461	348	779
	Temeke	1107	412	311	695
	**Total**	**4577**	**1703**	**1285**	**2874**
2011	Kinondoni	2311	860	649	1451
	Ilala	1258	468	353	790
	Temeke	1100	409	309	691
	**Total**	**4669**	**1737**	**1311**	**2932**
2012	Kinondoni	2205	970	733	1235
	Ilala	1200	528	399	672
	Temeke	992	436	330	556
	**Total**	**4397**	**1935**	**1461**	**2462**
2013	Kinondoni	2304	1106	835	1198
	Ilala	1340	643	486	697
	Temeke	1017	488	369	529
	**Total**	**4661**	**2237**	**1689**	**2424**
2014	Kinondoni	2205	942	734	1263
	Ilala	1399	597	466	802
	Temeke	1001	427	333	574
	**Total**	**4605**	**1966**	**1534**	**2639**
2015	Kinondoni	2174	809	631	1365
	Ilala	1391	517	404	874
	Temeke	1008	375	292	633
	**Total**	**4573**	**1701**	**1327**	**2872**
2016	Kinondoni	2250	837	653	1413
	Ilala	1480	551	429	929
	Temeke	1010	376	293	634
	**Total**	**4740**	**1763**	**1375**	**2977**
2017	Kinondoni	2600	1014	811	1586
	Ilala	1408	549	421	859
	Temeke	1250	488	361	763
	**Total**	**5258**	**2051**	**1593**	**3207**
